# Adrenal crises and quality of life after bilateral adrenalectomy vs primary autoimmune adrenal insufficiency

**DOI:** 10.1210/jendso/bvag104

**Published:** 2026-05-02

**Authors:** Alexandra N Bancos, Raul Gregg-Garcia, Natalia Genere, Sanan Mahrokhian, Sara J Achenbach, Elizabeth J Atkinson, Anand Vaidya, Irina Bancos

**Affiliations:** Division of Endocrinology and Metabolism, Department of Medicine, Mayo Clinic, Rochester, MN 55905, USA; Division of Endocrinology and Metabolism, Department of Medicine, Mayo Clinic, Rochester, MN 55905, USA; Department of Internal Medicine, Indiana University, Indianapolis, IN 46202, USA; Division of Endocrinology and Metabolism, Department of Medicine, Mayo Clinic, Rochester, MN 55905, USA; Division of Endocrinology, Diabetes, Bone and Mineral Disorders, Department of Medicine, Henry Ford Health, Detroit, MI 48202, USA; Center for Adrenal Disorders, Brigham and Women's Hospital, Division of Endocrinology, Diabetes, and Metabolism, Mass General Brigham, Harvard Medical School, Boston, MA 02115, USA; Division of Clinical Trials and Biostatistics, Department of Quantitative Health Sciences, Mayo Clinic, Rochester, MN 55905, USA; Division of Clinical Trials and Biostatistics, Department of Quantitative Health Sciences, Mayo Clinic, Rochester, MN 55905, USA; Center for Adrenal Disorders, Brigham and Women's Hospital, Division of Endocrinology, Diabetes, and Metabolism, Mass General Brigham, Harvard Medical School, Boston, MA 02115, USA; Division of Endocrinology and Metabolism, Department of Medicine, Mayo Clinic, Rochester, MN 55905, USA; Department of Laboratory Medicine and Pathology, Mayo Clinic, Rochester, MN 55905, USA

**Keywords:** AI, PAI, BLA, AA, AAI, AddiQoL, QoL, management

## Abstract

**Context:**

Bilateral adrenalectomy (BLA) and autoimmune adrenal insufficiency (AAI) are 2 major causes of primary adrenal insufficiency (PAI). Comparative data between etiologies are limited.

**Objective:**

To compare management, quality of life (QoL), and frequency of adrenal crises between participants post-BLA vs AAI.

**Methods:**

We conducted a dual-center, cross-sectional study of adults with AAI or BLA between 2018 and 2025. All participants completed questionnaires on management, adrenal crises, and the Addison Disease-specific QoL Questionnaire (AddiQoL).

**Results:**

Of 343 participants, 203 had AAI (median age 54 years, 72% women) and 139 had BLA (median age 57 years, 76% women). AddiQoL scores were similar between participants with AAI and BLA (median 84 vs 83, *P* = .947), but better in participants post-BLA for pheochromocytoma versus hypercortisolism (median 88 vs 81, *P* = .031). Participants with AAI reported a higher number of adrenal crises within the past year than those post-BLA (25% vs 13%, *P* = .022). In multivariable analyses of age, sex, glucocorticoid dose and type, PAI type, AddiQoL score, and autoimmune comorbidities, only PAI type was associated with higher number of reported adrenal crisis within the last year (AAI vs BLA: odds ratios of 2.1-2.2, *P* < .05 for all models).

**Conclusion:**

QoL was comparable between participants with AAI and BLA, though patients post-BLA for pheochromocytoma reported better QoL than patients post-BLA for hypercortisolism. Adrenal crises were more common in participants with AAI than those post-BLA, a finding that was not explained by patients’ glucocorticoid management, underscoring the need for tailored crisis prevention strategies.

Although quality of life (QoL) and outcomes in patients with primary adrenal insufficiency (PAI) have been a topic of interest in recent decades [1-15], comparisons between PAI subtypes remain scarce [16, 17]. Bilateral adrenalectomy (BLA), a seldomly performed procedure encompassing 4% of all adrenalectomies [18], leads to PAI necessitating life-long glucocorticoid and mineralocorticoid replacement therapy [19-22]. BLA may be indicated for treatment of bilateral pheochromocytomas, overt Cushing syndrome, bilateral adrenal metastases, congenital adrenal hyperplasia, or primary aldosteronism [23-26].

Limited evidence is available on the burden of disease, QoL, and adrenal crises in participants with PAI resulting from BLA. Large-scale studies reporting on QoL, adrenal crises, and burden of disease in participants with PAI rarely provided subgroup analysis based on PAI subtype [12-14]. Several studies that investigated participants with Cushing syndrome compared participants’ QoL before and after BLA, noting improvement [27-32]. However, QoL assessment did not always exclude participants within 12 months post-BLA [27, 28, 32], when participants were still recovering from the effects of Cushing syndrome and glucocorticoid withdrawal. Published studies in this area have also been limited by small cohort size, ranging between 11 and 40 participants [27-32]. Finally, QoL assessment did not include adrenal insufficiency-specific QoL questionnaires but instead used the 36-Item Short Form survey and Cushing syndrome-specific QoL questionnaires that do not capture a full range of symptoms associated with adrenal insufficiency.

Despite a shared diagnosis of PAI, patients post-BLA and patients with autoimmune adrenal insufficiency (AAI) differ in factors surrounding the initial diagnosis and associated comorbidities, which may impact QoL and frequency of adrenal crises. Patients with AAI usually experience a delay in diagnosis and may present with an adrenal crisis [33], whereas patients anticipated to develop PAI because of planned BLA undergo adrenal insufficiency education and become familiar with PAI management before the procedure. In addition, patients with AAI are at higher risk for development of other chronic autoimmune disorders that may complicate PAI management and impact QoL. In contrast, patients undergoing BLA for Cushing syndrome may take a long time to recover from the consequences of Cushing syndrome or its previous treatments, potentially complicating PAI management and QoL.

To address these gaps, we conducted a dual-center, cross-sectional study of participants with PAI resulting from BLA and AAI with the following objectives: (1) compare management, burden of disease, QoL, and adrenal crisis frequency in participants with PAI due to BLA vs AAI and (2) identify factors associated with differences in outcomes between the 2 cohorts.

## Methods

### Participants

The study protocol was approved at the Mayo Clinic (Rochester, MN) and at Brigham Women Hospital (Boston, MA). Participants were identified through a medical record search for patients with a diagnosis of adrenal insufficiency. Medical records were reviewed to confirm that PAI diagnosis was made by an adrenal endocrinologist. Etiology of PAI was determined based on the diagnosis documented in the medical record by an adrenal endocrinologist. In participants classified as having AAI, the diagnosis reflected the treating clinician's assessment based on biochemical confirmation of primary adrenal insufficiency and clinical context, including autoimmune comorbidities and/or presence of 21-hydroxylase autoantibodies when available. Participants with PAI resulting from BLA were only included if they were at least 1 year post-BLA to ensure recovery from glucocorticoid withdrawal. Participants were contacted via electronic communication/during routine endocrinological visits and signed a Health Insurance Portability and Accountability Act release form before participation. Participants then completed a baseline questionnaire and periodically received follow-up questionnaires. These included an annual Depression, Anxiety, and Stress Scale (DASS-21) questionnaire, annually alternating Addison Disease-specific Quality of Life (AddiQoL) questionnaire and Short Form-36 Health Survey, and a yearly follow-up questionnaire on symptoms, adrenal crises, burden of disease, and management. All participants had been evaluated at least once at Mayo Clinic or Brigham and Women's Hospital; however, routine longitudinal care for adrenal insufficiency may have been provided at other institutions and was not systematically captured in this study.

**Figure 1:**
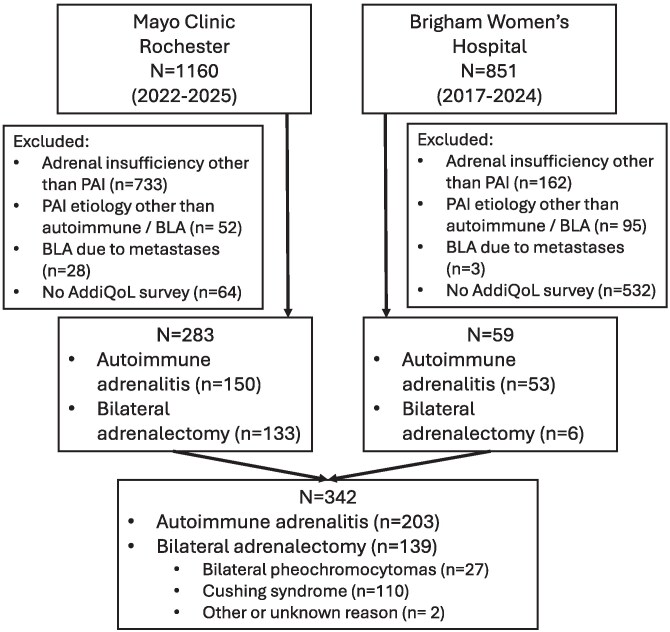
Recruitment flowchart.

This substudy is a cross-sectional analysis of adult participants with confirmed PAI resulting from AAI or BLA and who completed the baseline questionnaire and at least 1 AddiQol questionnaire. Participants with BLA resulting from metastases were excluded. Reasons for BLA were abstracted from the medical record and included Cushing syndrome (ACTH dependent and independent, or unknown), bilateral pheochromocytomas, or other (Fig. 1).

### Baseline and follow-up questionnaire

The baseline questionnaire contained the following sections: demographic information, circumstances around initial diagnosis, prediagnosis and postdiagnosis symptoms of adrenal insufficiency, current comorbidities, adrenal insufficiency management, adrenal insufficiency education, and adrenal crisis frequency and response. Replacement glucocorticoid and mineralocorticoid doses were obtained from the same questionnaire that included the AddiQoL survey used for analysis, whether completed at baseline or during follow-up. This approach ensured that treatment variables corresponded to the time point at which QoL outcomes were assessed. Outcomes were reported either as events occurring “since diagnosis” or “within the past year,” depending on the questionnaire item. We ensured accuracy of demographic and diagnosis information based on a medical record review, but the remaining questions were participant reported. To compare total daily glucocorticoid therapy, we converted therapies other than hydrocortisone into hydrocortisone (1 mg prednisone/prednisolone = 5 mg hydrocortisone equivalents, 1 mg dexamethasone = 60 mg hydrocortisone equivalents).

Questions about relevant clinical parameters were determined by the investigators of the study and input from a panel of participants with AI [12]. Relevant clinic parameters included overreplacement or underreplacement symptoms, comorbidities, number of adrenal crises since diagnosis and within the past year, number and reason of stress dose glucocorticoid usage in the past year, extent of participant education (having injectable glucocorticoids at home and comfort level with usage, wearing medical alert gear, and having an adrenal action plan for tailored stress responses), and extent of support in management (medical insurance support, financial impact caused by AI, support from family and friends, and difficulty with self-management).

### Validated questionnaires

The 30-question AddiQoL questionnaire measures QoL markers in 4 adrenal insufficiency-specific subdimensions: adrenal insufficiency Symptoms, Fatigue, Emotions, and Miscellaneous (sleep, sex life, and intercurrent sickness). AddiQoL provides a total score between 0 and 120 and higher scores indicate a better QoL [11].

The DASS-21 is a validated questionnaire for measuring the effects of depression, anxiety, and stress on a participant's life. DASS-21 provides a score between 0 and 42 within 3 categories: depression, anxiety, and stress. Higher scores indicate more severe effects on daily life [34, 35]. The DASS-21 instrument includes established severity thresholds derived from population reference values (severe depression ≥ 11, severe anxiety ≥ 10, severe stress ≥ 17); however, in this study the scores were used primarily to compare psychological symptom burden between PAI subtypes.

### Adrenal crisis definition

Adrenal crises were determined by asking participants to self-report the number of times they have gone to an emergency room for an adrenal crisis. Baseline questionnaires asked for number of adrenal crises since diagnosis, whereas yearly follow-up questionnaires asked for number of adrenal crises within the past year. Other questions related to adrenal crises included (1) the participant's experience at the emergency department/hospital, (2) who supports the participant during adrenal crises, and (3) whether the participant has injectable steroids at home and how often the injectable steroids are used.

### Statistical analysis

Continuous variables were summarized as median and interquartile range (IQR) and groups were compared using the Wilcoxon rank-sum test. Categorial variables were summarized as frequencies and percentages and groups were compared using the chi-squared test. Pearson correlations and linear regression models were used to explore associations with AddiQoL score. Factors that were assessed in the multivariable linear regression models included age, female sex, supportive insurance, daily hydrocortisone equivalent, prednisone, duration of adrenal insufficiency, possession of medical alert bracelet or at-home injectables, and adrenal action plan. Logistic regression models were used to examine associations with having an adrenal crisis in the past year. All models were predefined. All the statistical analyses were conducted using SAS version 9.4 (SAS Institute, Cary, NC, USA) and R 4.4.1 (R Foundation for Statistical Computing, Vienna, Austria). *P* < .05 was defined as statistically significant.

The most recent AddiQoL and DASS-21 questionnaires filled out by participants were used in analyses. Missing components of the AddiQoL questionnaire were imputed using the predictive mean matching algorithm in the R package *mice* [36] before calculation of the total AddiQol score.

Analyses on adrenal crises within the past year were conducted on follow-up questionnaire responses. Participants without follow-up surveys were still included if their baseline survey was completed less than 2 years following diagnosis, in which case the reported number of adrenal crises since diagnosis were the same as those in the past year, or if their baseline survey reported zero adrenal crises since diagnosis, in which the reported number of adrenal crises within the past year was also zero.

## Results

### Baseline characteristics

Of the 342 participants with PAI, 203 were diagnosed with AAI and 139 were post-BLA (Fig. 1). Median age at the time of study participation was 56 (IQR 43-67) years, and 251 (73%) participants were women, without significant group differences. Participants with AAI had a longer duration of PAI than participants with BLA (median of 14 years [IQR 7-28] vs median of 10 years [IQR 5-21], *P* = .008). As expected, the AAI cohort had a higher prevalence of autoimmune comorbidities while the BLA cohort had a higher prevalence of cardiovascular comorbidities (Table 1).

**Table 1:** Demographics and adrenal insufficiency management in patients with primary adrenal insufficiency

Variable	N available	Autoimmune adrenal insufficiency (n = 203)	Bilateral adrenalectomy*^a^* (n = 139)	*P* value
Demographic data				
Women, n (%)	342	146 (72)	105 (76)	.457
Non-Hispanic White, n (%)	327	182 (96)	121 (88)	.**011**
Age at time of survey, years, median (IQR)	342	54 (42-67)	57 (44-67)	.426
Age at diagnosis, years, median (IQR)	342	35 (23-47)	39 (29-53)	.**004**
Duration of adrenal insufficiency, years, median (IQR)	342	14 (7-28)	10 (5-21)	.**008**
Adrenal insufficiency management				
Glucocorticoid type, n (%)	342			
Hydrocortisone alone		163 (80)	107 (77)	.254
Prednisone/prednisolone alone		30 (15)	29 (21)	
Hydrocortisone + prednisone		7 (3)	1 (1)	
Dexamethasone alone		2 (1)	2 (1)	
Dexamethasone + prednisone		1 (1)	0 (0)	
Daily hydrocortisone equivalent dose, mg, median (IQR)	339	21 (20-28)	20 (20-25)	.221
Frequency of glucocorticoid daily dosing, n (%)	334			.**023**
Once daily		31 (16)	31 (22)	
Twice daily		103 (53)	81 (58)	
Three times daily		62 (32)	26 (19)	
Fludrocortisone, n (%)	337	183 (92)	128 (93)	.788
Fludrocortisone dose reported, n (%)	308			
Less than 0.05 mg/day		31 (17)	17 (13)	.218
0.05 mg/day		50 (28)	42 (33)	
0.10 mg/day		79 (44)	62 (48)	
More than 0.10 mg/day		20 (11)	7 (5)	
Dehydroepiandrosterone supplement (women only)	251	23 (16)	17 (16)	.926
Has injectable glucocorticoid, n (%)	340	170 (85)	115 (83)	.650
Moderate/high comfort level with injecting glucocorticoid, n (%)	341	135 (67)	95 (69)	.651
Has medical alert bracelet/necklace, n (%)	339	164 (81)	114 (83)	.634
Has an adrenal action plan	283	81 (54)	64 (48)	.323
Comorbidities				
Autoimmune comorbidities, n (%)	342			
Hypothyroidism		91 (45)	19 (14)	**<**.**001**
Hyperthyroidism		19 (9)	10 (7)	.480
Type 1 diabetes		18 (9)	1 (1)	.**001**
Pernicious anemia		17 (8)	3 (2)	.**016**
Vitiligo		15 (7)	0 (0)	.**001**
Celiac disease		9 (4)	1 (1)	.**045**
Cardiovascular comorbidities, n (%)	342			
Dyslipidemia		50 (25)	28 (20)	.331
Hypertension		21 (10)	29 (21)	.**007**
Type 2 diabetes		8 (4)	16 (12)	.**007**
Congestive heart failure		3 (1)	5 (4)	.203
Cerebrovascular accident		1 (0)	5 (4)	.**032**
Atrial fibrillation		2 (1)	3 (2)	.375
Myocardial infarction		1 (0)	3 (2)	.159
Osteoporosis/osteopenia	342	42 (21)	37 (27)	.201

Bolded values indicate statistically significant results.

Abbreviation: IQR, interquartile range.

^*a*^Reasons for bilateral adrenalectomy included: pituitary Cushing syndrome (62, 45%), adrenal Cushing syndrome (29, 21%), bilateral pheochromocytomas (27, 19%), ectopic Cushing syndrome (14, 10%), Cushing syndrome, unknown type (5, 4%), bilateral adenoma (1, 1%), and unknown reason (1, 1%).

### Circumstances surrounding initial AI diagnosis

The majority of participants with AAI reported at least 1 visit to the emergency department (119, 59%) and having to see at least 2 physicians (177, 88%), with 25% reporting seeing at least 5 physicians, to receive the initial diagnosis of PAI. In addition, 139 (69%) reported it took at least 1 year after onset of symptoms to receive a diagnosis, with 31 (16%) reporting more than 5 years of symptoms before diagnosis, Table S1 [37].

Participants with PAI resulting BLA were treated for pituitary Cushing syndrome (62, 45%), ACTH-independent Cushing syndrome or mild autonomous cortisol secretion (29, 21%), bilateral pheochromocytomas (27, 19%), ectopic Cushing syndrome (14, 10%), unknown Cushing syndrome type (5, 4%), bilateral adenoma (1, 1%), and 1 (1%) unknown reason (Table 1).

### Management of PAI

There were no differences in glucocorticoid or mineralocorticoid replacement therapy between participants with AAI vs participants post-BLA, with the majority treated with hydrocortisone (80% vs 77%), prednisone/prednisolone (15% vs 21%), and other regimens (5% vs 2%). Daily median hydrocortisone equivalent dose was similar in both groups (median of 21 vs 20 mg, *P* = .221). Likewise, there was no difference in fludrocortisone use or dose between groups (Table 1). Most participants had a medical alert bracelet/necklace (AAI vs BLA: 81% vs 83%, *P* = .634) and injectable glucocorticoids (AAI vs BLA: 85% vs 83%, *P* = .650) with most reporting a moderate to high comfort level with self-injecting (AAI vs BLA: 67% vs 69%, *P* = .651). Approximately half of participants had an adrenal action plan (AAI vs BLA: 54% vs 48%, *P* = .323) which provided patient-specific guidelines for managing adrenal insufficiency during times of stress (Table 1).

### Current symptoms and perceived burden of disease

**Figure 2:**
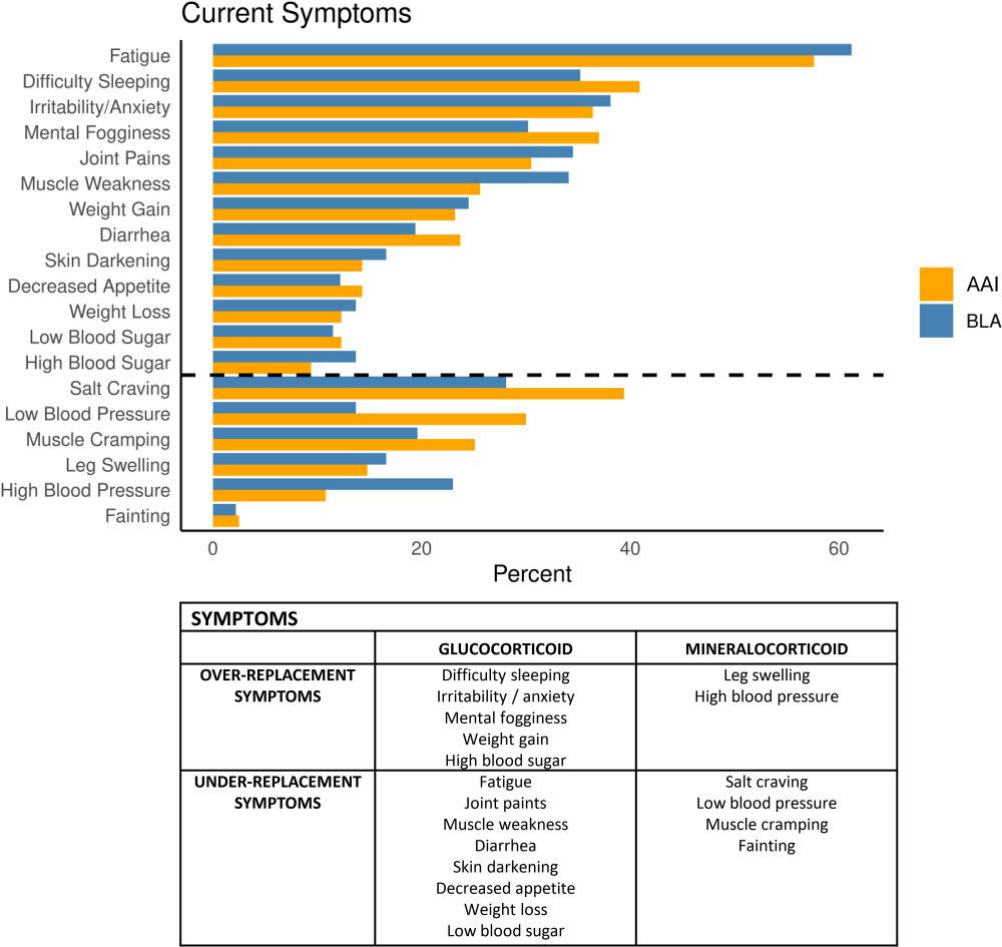
Self-reported symptoms of under- or overglucocorticoid replacement (above dashed line) and mineralocorticoid replacement (below dashed line).

**Figure 3:**
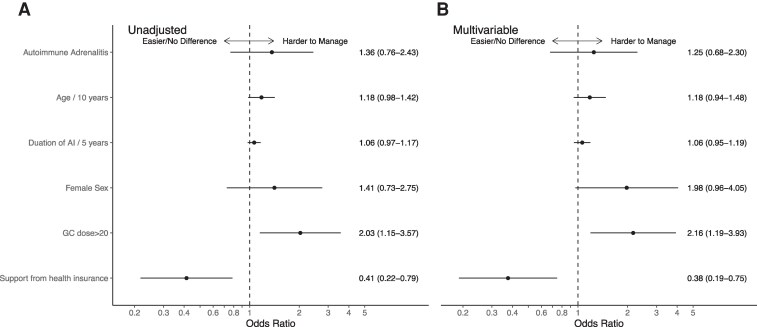
Factors associated with harder to manage adrenal insufficiency since the initial diagnosis, both unadjusted (A) and adjusted for age and sex (B).

Overall, participants in both groups reported a similar number of symptoms related to PAI management. However, participants with AAI reported a higher number of mineralocorticoid underreplacement symptoms (51% vs 35% in BLA, *P* = .001), despite a similar management with fludrocortisone (Table S2 [37], Fig. 2). No differences in exercise, limitations of daily activities, insurance support, or financial burden were found between participants with AAI vs BLA, although the majority reported some impact on their ability to exercise and engage in daily life activities (Table S2 [37]). Factors associated with perceived harder-to-manage PAI included less support from health insurance and taking a higher daily hydrocortisone equivalent dose (>20 mg/day) (Fig. 3).

**Figure 4:**
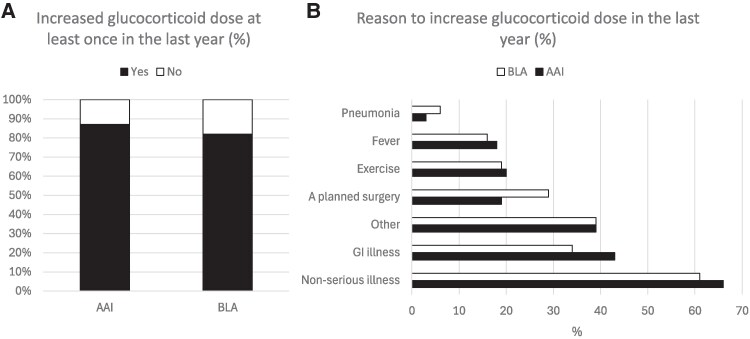
Percentage of cohort that reported increasing their glucocorticoid dose at least once in the past year (A) and self-reported reasons for increasing (B).

Most participants reported increasing their glucocorticoid dose at least once in the past year (AAI vs BLA: 87% vs 82%, *P* = .193), and one third of participants reported increasing more than 5 times (37% vs 35%, *P* = .794). Glucocorticoids were most commonly increased for a nonserious illness, gastrointestinal illness, and an unspecified “other” category. Of those who increased their dose, only about 20% of participants did so for exercise (Fig. 4).

### Quality of life

Participants with AAI and BLA had similar total AddiQoL scores (median of 84 vs 83, *P* = .947), only differing in the miscellaneous (sleep, sex life, and intercurrent disease) category, which was slightly lower in participants with AAI vs BLA (median of 11 vs median of 12, *P* = .002) (Table 2). No differences in the DASS-21 scores were noted in participants with AAI vs BLA (Table 2). Comparing participants with BLA for bilateral pheochromocytoma versus BLA for Cushing syndrome, those with BLA for bilateral pheochromocytoma had a better total AddiQoL score (median of 88 vs 81, *P* = .031), as well as better subscores in adrenal insufficiency symptoms (median of 29 vs 27, *P* = .030) and fatigue (median of 23 vs 21, *P* = .013) subdimensions (Table S3 [37]). After adjusting for age, sex, and duration of adrenal insufficiency, total AddiQoL score remained higher (better) in patients treated with BLA for pheochromocytoma vs those treated with BLA for Cushing syndrome (*R* = 0.20, *P* = .018).

**Table 2:** AddiQoL and DASS scores in patients with primary adrenal insufficiency

Variable	N available	Autoimmune adrenal insufficiency (n = 203)	Bilateral adrenalectomy (n = 139)	*P* value
AddiQoL, median (IQR)				
Total AddiQol score (maximum possible = 120)	341	84 (75-93)	83 (74-95)	.947
Adrenal insufficiency symptoms (maximum possible = 36)		28 (25-31)	28 (24-31)	.121
Emotion (maximum possible = 32)		23 (21-26)	24 (21-26)	.543
Fatigue (maximum possible = 32)		22 (17-25)	21 (17-25)	.800
Miscellaneous (maximum possible = 20)		11 (9-13)	12 (10-14)	.**002**
DASS, median (IQR)				
DASS total score (maximum possible = 63)	305	9 (4-15)	8 (4-18)	.890
DASS depression (maximum possible = 21)	321	2 (0-5)	2 (1-6)	.478
DASS anxiety (maximum possible = 21)	327	2 (1-6)	2 (1-5)	.644
DASS stress (maximum possible = 21)	329	4 (1-7)	3 (1-6)	.847

Bolded value indicate statistically significant results.

Abbreviations: AddiQoL, Addison Disease-specific QoL Questionnaire; DASS, Depression Anxiety Stress Scale; IQR, interquartile range.

In the overall cohort, after adjusting for age and sex, factors associated with a higher (better) AddiQoL score included older age, male sex, lower glucocorticoid dose, hydrocortisone therapy, better DASS-21 scores, active employment, and a supportive insurance (Table S4, S5 [37]). In the multivariable model, older age, male sex, supportive insurance, lower daily hydrocortisone equivalent dose, and hydrocortisone (over prednisone) therapy were all independent factors associated with a higher AddiQoL score (Table 3). Older age was no longer associated with higher AddiQoL scores when accounting for longer duration of disease. Interestingly, participants with a medical alert bracelet or available glucocorticoid injectable had a lower AddiQoL score than those without (Table 3). However, this was no longer associated with worse QoL when accounting for the presence of an adrenal action plan (a patient-specific guideline for managing adrenal insufficiency during times of stress) (Table 3).

**Table 3:** Multivariable linear regression model of factors associated with higher (better) AddiQoL score

Variable	Model 1		Model 2		Model 3		Model 4	
	β estimate (95% CI)	*P* value	β estimate (95% CI)	*P* value	β Estimate (95% CI)	*P* value	β Estimate (95% CI)	*P* value
Age (per 10-year increase)	1.0 (0.1-2.0)	.**033**	0.8 (−0.3 to 1.9)	.152	1.2 (0.2- 2.1)	.**018**	1.3 (0.2- 2.4)	.**021**
Women (vs men)	−6.7 (−10.1 to −3.4)	**<**.**001**	−6.0 (−9.4 to −2.6)	**<.001**	− 6.5 (−9.8 to −3.1)	**<**.**001**	−6.8 (−10.6 to −2.9)	**<**.**001**
Supportive insurance (vs not supportive)	6.0 (2.1- 9.9)	.**003**	6.5 (2.6- 10.4)	**.001**	6.7 (2.8- 10.6)	**<**.**001**		
Daily hydrocortisone equivalent (per 5 mg/day increase)	−1.8 (−2.8 to −0.8)	**<**.**001**	−1.7 (−2.7 to −0.7)	**<.001**	−1.7 (−2.7to −0.7)	.**001**	−2.4 (−3.5 to −1.2)	**<**.**001**
Prednisone (vs hydrocortisone therapy)	—	—	−4.7 (−8.2 to −1.1)	**.011**	−4.7 (−8.2 to −1.1)	.**011**		
Duration of adrenal insufficiency (per 5-year increase)	—	—	0.4 (−0.2 to 1.0)	.169	—	—		
Have medical alert bracelet or at-home injectables	−7.9 (−15.0 to −0.8)	.**029**	—	—	—	—	−6.1 (−16.7 to 4.6)	.266
Has adrenal action plan							−0.6 (−3.9 to 2.7)	.721

Bolded values indicate statistically significant results.

Abbreviation: AddiQoL, Addison Disease-specific QoL Questionnaire.

### Adrenal crises

A higher number of participants with AAI reported experiencing at least 1 adrenal crisis since diagnosis (65 vs 51%, *P* = .010), as well as within the past year (25% vs 13%, *P* = .022), when compared to participants post-BLA (Table 4). Likewise, the median number of adrenal crises per person-years was higher in participants with AAI (0.12 vs 0.02 in BLA, *P* = .009) (Table 4).

**Table 4:** Adrenal crises in patients with primary adrenal insufficiency

Variable	N available	Autoimmune adrenal insufficiency (n = 203)	Bilateral adrenalectomy (n = 139)	*P* value
Had at least 1 adrenal crisis within the past year, n (%)	261	41 (25)	13 (13)	.**022**
Had at least 1 adrenal crisis since diagnosis, n (%)	340	132 (65)	70 (51)	.**010**
Number adrenal crises/100 person-years, median (IQR)	336	12 (0-27)	2 (0-30)	.**009**
Rate categories, n (%)				
0		65 (32%)	67 (49%)	.**014**
1-49		105 (52%)	50 (37%)	
50-99		21 (10%)	11 (8%)	
≥100		10 (5%)	7 (5%)	
Times used injectable glucocorticoids for an adrenal crisis in the past year, n (%)	158			
0		96 (88)	39 (80)	.536
1		9 (8)	7 (14)	
2		2 (2)	1 (2)	
≥3		2 (2)	2 (4)	
Experience at the emergency department in the past year, n (%)	60			
Received prompt treatment with glucocorticoids		20 (45)	5 (31)	.465
Received prompt treatment with glucocorticoids only after patient/family explained diagnosis		14 (32)	5 (31)	
Emergency personnel did not understand my problem and my treatment was delayed		10 (23)	6 (38)	

Bolded values indicate statistically significant results.

**Figure 5:**
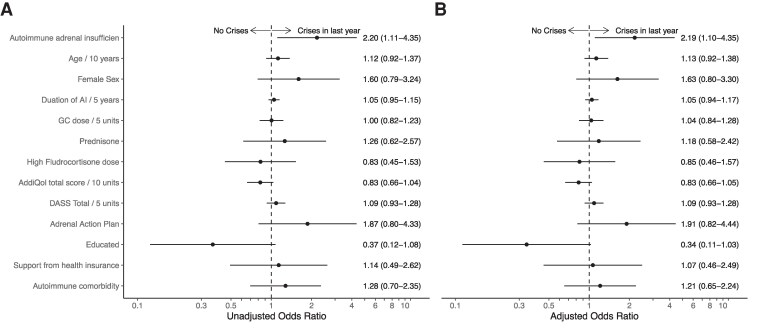
Factors associated with adrenal crisis development in the past year based on self-reporting with (A) demonstrating unadjusted analysis and (B) demonstrating age- and sex-adjusted analysis.

AAI (vs BLA) was associated with approximately twice the number of adrenal crises within the past year even after adjusting for age, sex, duration of adrenal insufficiency, daily glucocorticoid dose, glucocorticoid type, AddiQoL score, PAI subtype, and presence of autoimmune comorbidities (Table 5, Fig. 5). However, after accounting for the presence of an adrenal action plan before survey completion, PAI subtype was no longer associated with adrenal crisis within the past year (Table 5).

**Table 5:** Multivariable logistic regression model of factors associated with adrenal crises within the past year

Variable	Model 1 OR (95% CI)	Model 2 OR (95% CI)	Model 3 OR (95% CI)	Model 4 OR (95% CI)	Model 5 OR (95% CI)
Autoimmune adrenal insufficiency (vs bilateral adrenalectomy)	**2.1 (1.1-4.3)**	**2.1 (1.1-4.2)**	**2.2 (1.1-4.4)**	**2.2** (**1.0-4.5)**	1.7 (0.8-4.0)
Age (per 10-year increase)	1.1 (0.9-1.4)	1.1 (0.9-1.4)	1.1 (0.9-1.4)	1.1 (0.9-1.4)	1.1 (0.9-1.5)
Women (vs men)	1.6 (0.8-3.4)	1.5 (0.7-3.2)	1.4 (0.6-2.9)	1.6 (0.8-3.3)	
Duration of adrenal insufficiency (per 5-year increase)	1.0 (0.9-1.1)				
Daily glucocorticoid dose (hydrocortisone equivalent (per 5-mg/day increase)	1.0 (0.8-1.3)	1.0 (0.8-1.3)	1.0 (0.8-1.2)	1.0 (0.8-1.3)	
Prednisone (vs hydrocortisone therapy)		1.1 (0.5-2.4)			
AddiQoL score (per 10-point increase)			0.8 (0.6-1.0)		
Autoimmune comorbidity of Graves’ disease, Hashimoto disease, type 1 diabetes, celiac disease, or pernicious anemia				1.0 (0.5-1.9)	
Has adrenal action plan*^a^*					1.9 (0.8-4.4)

Statistically significant results are bolded.

^a^Available only for n = 202 participants (Mayo Clinic site) of the 261 who had information available about adrenal crises within the past year.

## Discussion

In this cross-sectional study examining differences between participants with PAI resulting from AAI vs BLA, we showed that management, burden of disease, and QoL are similar between the groups. However, QoL in patients with BLA for pheochromocytoma was better than QoL in patients with BLA for Cushing syndrome. Despite similarities in management and QoL between AAI and BLA cohorts, participants with AAI reported a near 2-fold increase in adrenal crises when compared to BLA—an unexpected finding.

### Management

We found no differences in glucocorticoid and mineralocorticoid management, medical bracelet wear, availability of injectable glucocorticoid or the comfort level of injecting glucocorticoids, and the presence of an adrenal action plan between participants with AAI and BLA. The majority of participants were treated with hydrocortisone with a median daily dose of 20 mg hydrocortisone equivalents, consistent with the guidelines for adrenal insufficiency management [38, 39], and concordant with some [5, 12] but not all [2, 11] previously published reports. Although the total number of reported symptoms related to glucocorticoid and mineralocorticoid therapy was similar between the 2 groups, participants with AAI reported a higher number of symptoms reflective of mineralocorticoid underreplacement (salt craving and hypotension). The reason for this seems unrelated to glucocorticoid and mineralocorticoid therapy as no differences in the glucocorticoid and mineralocorticoid dose were found.

### Quality of life

In this study, participants with AAI and BLA had similar AddiQoL scores except for a minor difference in the miscellaneous AddiQoL subscore (reflective of sleep, sexual satisfaction, and impact of intercurrent disease), which was better in participants post-BLA. Both total AddiQoL scores and subscores in our study are concordant with those reported in the literature for participants with PAI [2, 8-11]. In our study, we were also able to perform a subgroup analysis of QoL based on the reason for BLA, finding a better quality of life in participants who had BLA for bilateral pheochromocytomas than those who had BLA to treat Cushing syndrome, even after adjusting for demographic differences and duration of PAI. This may reflect the lasting impact of previous hypercortisolism on current quality of life, as well as potential associated morbidity of other treatments for Cushing syndrome.

Notably, we found that younger participants and women had worse QoL, although age was no longer a significant factor when adjusting for the duration of PAI. A lower QoL in women has been consistently reported in previous studies conducted in participants with PAI [2, 5, 7, 9, 11]. In contrast, our finding of worse QoL in younger participants is discordant with other studies that report worse QoL scores in older individuals [2, 7, 9, 11]. However, these studies did not consider the impact of PAI duration, in addition to age.

We found that a higher total daily glucocorticoid dose was associated with worse QoL. Interestingly, the effect of glucocorticoid dosage on QoL is discrepant among current literature, with some studies showing no association [2, 5, 11], whereas other studies reporting a worse QoL in participants treated with higher daily glucocorticoids [1, 4]. We also found that prednisone (vs hydrocortisone) was associated with worse QoL, though the glucocorticoid type did not impact the risk of adrenal crisis. In contrast to this finding, we previously showed that participants with postoperative adrenal insufficiency and history of Cushing syndrome reported better QoL when treated with prednisone (as opposed to hydrocortisone) during the 3 month postoperative period [40]. Of note, this population of participants also suffered from glucocorticoid withdrawal; thus, these findings may not be applicable to participants with chronic PAI. Because hydrocortisone is the usual therapy for participants with PAI, it is possible that the lower QoL we noticed with prednisone was associated not with the glucocorticoid type itself, but some confounding factor that required these participants to transition from the standard hydrocortisone therapy to an alternative.

Last, we found that owning a medical alert bracelet/at-home injectable glucocorticoid was associated with a worse QoL. This was no longer significant after accounting for the presence of an adrenal action plan (a patient-specific guideline for managing adrenal insufficiency during times of stress), potentially reflective of the importance of patient education (and physical documentation of stress-dose requirements).

### Adrenal crises

We found that participants with AAI reported a higher number of adrenal crises (for which they visited the emergency department or hospital) both since diagnosis and in the past 1 year. This was a surprising finding because no differences in management and education on PAI were reported by participants with AAI vs BLA. Multivariable analyses of age, sex, duration of PAI, daily glucocorticoid dose, glucocorticoid type, AddiQoL score, PAI subtype, and presence of autoimmune comorbidities again demonstrated that only PAI subtype (AAI vs BLA) was associated with a 2-fold increased risk in adrenal crises. This association was no longer significant in a subgroup analysis that included presence of an adrenal action plan (a patient-specific guideline for managing adrenal insufficiency during times of stress). These results may be a reflection of a lower sample size in this analysis or may indicate that having patient-specific education minimizes differences between PAI subtypes associated with adrenal crisis development.

Few studies examined differences in adrenal crises between patients with AAI vs BLA. In a smaller study of 200 patients with PAI in Belgium, no difference was found between adrenal crises in AAI (n = 125) versus BLA (n = 47) cohorts [17]. The same lack of association with PAI subtype was found in a study of 113 women with PAI (including 50 with AAI and 6 post-BLA) during pregnancy [16]. In noncomparative studies, incidence of adrenal crisis in patients with AAI and BLA varied based on study, but remained between 3 and 10 per 100 participant-years for both groups [15, 26, 29].

Adrenal crisis most commonly develops during periods of physical or emotional stress such as infections, hospital procedures, or other sickness, when glucocorticoids are not adequately replaced [40-45]. Reported risk factors for adrenal crisis include a higher glucocorticoid daily dose [46], longer duration of PAI [47], history of a previous adrenal crisis [41, 48], and deficits in both patient and clinician education [49]. Previous studies have shown that certain autoimmune comorbidities are also associated with an increased risk for adrenal crises. In 1 study, participants with PAI resulting from polyglandular syndrome and those with PAI and type 1 diabetes mellitus reported more adrenal crises than participants with isolated PAI without associated autoimmune comorbidities [15]. This positive association between comorbidities and higher risk for adrenal crises was also reported in other studies on adrenal insufficiency [42, 44, 45].

In our study, presence of autoimmune comorbidities was not associated with adrenal crisis frequency. We did find that emergency department management of adrenal crises was suboptimal with only 31% to 45% of participants receiving prompt treatment with glucocorticoids when visiting the emergency department. However, no differences in emergency department treatment of participants with AAI vs BLA were found to explain a higher prevalence of adrenal crises in AAI. Likewise, we could not find associations with daily glucocorticoid dose, duration of PAI, or patient education based on the presence of an adrenal action plan. However, our AAI cohort did report more adrenal crises since diagnosis, putting them at increased risk for a second adrenal crisis based on previous studies [41, 48].

### Strengths and limitations

Strengths of our study include a large sample size, inclusion of BLA cohort at least 1 year postsurgery (avoiding the period of glucocorticoid withdrawal), in-depth questionnaire, and use of a validated PAI-specific QoL questionnaire. Furthermore, we were able to perform a subgroup analysis based on reason for BLA.

Limitations include referral bias, information bias, reporting bias, and recall bias. In addition, our study had a predominance of a White, non-Hispanic population, limiting generalizability to more diverse populations. This study was conducted in United States; thus, some of the experience captured may be unique to the US medical system. In this study, we were not able to capture socioeconomic status of participants, which may have an impact on the QoL and adrenal crises. We also observed that a small proportion of participants reported once-daily hydrocortisone use, likely reflecting heterogeneity in real-world glucocorticoid replacement strategies as well as limitations of self-reported survey data. Definition of adrenal crisis was based on self-reporting and the wording of our question—“how many times have you gone to the emergency department for an adrenal crisis”—which could have resulted in both over- and underreporting. Importantly, adrenal crisis does not have a universally applied definition in clinical practice, and patients may be labeled as having an adrenal crisis even when strict criteria are not fulfilled. In many published definitions [50-52], adrenal crisis is characterized by acute clinical deterioration with hypotension and rapid improvement following parenteral glucocorticoid administration. Because our study relied on patient-reported emergency room visits rather than adjudicated clinical events, it is possible that some reported episodes did not meet these criteria, leading to potential misclassification. Additionally, frequency of adrenal crises within the past 1 year could have been underestimated in some participants due to a gap >1 year between surveys. Importantly, there are many confounding factors in our analysis of adrenal crises. Presumably, not all participants go to the emergency department for an adrenal crisis, choosing to use their at-home injectable glucocorticoid alone. Others may go to the emergency department for an “adrenal crisis” that may have not met clinical criteria for severity. Because of this subjectivity, it is possible that the higher number of adrenal crises in AAI cohort could be due to a higher sensitivity toward symptoms or fear of adrenal crisis, possibly as a result of delays in PAI diagnosis, prolonged unexplained symptomatology before diagnosis, and, in some, initial diagnosis resulting from an adrenal crisis. It is also possible that those with BLA were more extensively coached on management requirements for PAI as a result of their planned surgical procedure—gaining practice in stress dosing postsurgery while on a glucocorticoid taper—whereas those with AAI were prescribed medication and educated only verbally, without the accompanying first-hand experience in managing their dose because of stress levels immediately following diagnosis.

## Conclusion

In conclusion, participants with PAI resulting from AAI vs BLA demonstrate no differences in their management, PAI education, burden of disease, and QoL. Participants with BLA for pheochromocytomas reported a better QoL than participants with BLA performed for Cushing syndrome. Surprisingly, despite very similar management, participants with AAI reported a 2-fold higher frequency of adrenal crises when compared to participants with BLA, underscoring the need for tailored crisis prevention strategies.

## Data Availability

Some or all datasets generated during and/or analyzed during the current study are not publicly available but are available from the corresponding author on reasonable request.

## Abbreviations

AAI: autoimmune adrenal insufficiency
AddiQoL: Addison Disease-specific QoL Questionnaire
BLA: bilateral adrenalectomy
DAAS-21: Depression, Anxiety, and Stress Scale
IQR: interquartile range
PAI: primary adrenal insufficiency
QoL: quality of life
